# 878. Contrast Is Not the Culprit: A Review of Acute Kidney Injury (AKI) in Burn Patients

**DOI:** 10.1093/jbcr/irag033.364

**Published:** 2026-04-06

**Authors:** Monica Hutson, Michael Erickson

**Affiliations:** The University of Texas Medical Branch Blocker Burn Unit, Galveston, TX; Blocker Burn Unit, Kemah, TX

## Abstract

**Introduction:**

Acute kidney injury (AKI) is a significant complication in burn patients, associated with increased morbidity and mortality. While contrast-enhanced imaging is often essential in trauma care, concerns about contrast-induced nephropathy (CIN) can limit its use. This study examines the occurrence of AKI in burn patients, considering not only contrast exposure but also injury severity, pre-existing illness, and resuscitation practices.

**Methods:**

A retrospective chart review of burn patients who required fluid resuscitation at a burn center was performed. Resuscitation was managed using a clinical decision support tool that provided real-time, patient-specific fluid recommendations, aiming to optimize outcomes while minimizing the risks of under-resuscitation and fluid overload.

**Results:**

Of the 50 patients reviewed, 10 underwent contrast-enhanced imaging, with 4 developing AKI and 6 remaining unaffected. Notably, an additional 6 patients who did not receive contrast also developed AKI. Patients who developed AKI (n = 10) were older, had more extensive burns, more co-morbidities, more frequent inhalation injuries, and required greater fluid resuscitation. AKI was associated with significant morbidity, with 4 patients requiring Continuous Renal Replacement Therapy (CRRT) and 4 deaths observed. Documented AKI etiologies included present on admission (n = 4), vancomycin-associated (n = 2), and multifactorial causes (n = 4).

**Conclusions:**

This study suggests that AKI in burn patients is multifactorial and not solely attributable to contrast exposure. The use of nephrotoxic medications, pre-existing renal compromise, infection, and hemodynamic instability play equally or more significant roles in AKI development. Avoiding contrast-enhanced imaging may therefore be an overly cautious approach that risks delaying essential diagnostic evaluations.

**Applicability of Research to Practice:**

This study highlights the importance of a comprehensive assessment when evaluating renal risk in burn patients. Contrast-enhanced imaging should not be withheld when clinically necessary, provided renal risk is managed through careful monitoring, minimization of nephrotoxins, and optimized resuscitation.

**Funding for the study:**

N/A.

